# A method for managing re-identification risk from small geographic areas in Canada

**DOI:** 10.1186/1472-6947-10-18

**Published:** 2010-04-02

**Authors:** Khaled El Emam, Ann Brown, Philip AbdelMalik, Angelica Neisa, Mark Walker, Jim Bottomley, Tyson Roffey

**Affiliations:** 1 Children's Hospital of Eastern Ontario Research Institute, 401 Smyth Road, Ottawa, Ontario K1J 8L1, Canada; 2 Pediatrics, Faculty of Medicine, University of Ottawa, Ottawa, Ontario, Canada; 3 GIS Infrastructure, Office of Public Health Practice, Public Health Agency of Canada, Ottawa, Ontario K1A 0K9, Canada; 4 Ottawa Hospital Research Institute, Ottawa, Ontario, Canada; 5 Children's Hospital of Eastern Ontario, 401 Smyth Road, Ottawa, Ontario K1J 8L1, Canada

## Abstract

**Background:**

A common disclosure control practice for health datasets is to identify small geographic areas and either suppress records from these small areas or aggregate them into larger ones. A recent study provided a method for deciding when an area is too small based on the uniqueness criterion. The uniqueness criterion stipulates that an the area is no longer too small when the proportion of unique individuals on the relevant variables (the quasi-identifiers) approaches zero. However, using a uniqueness value of zero is quite a stringent threshold, and is only suitable when the risks from data disclosure are quite high. Other uniqueness thresholds that have been proposed for health data are 5% and 20%.

**Methods:**

We estimated uniqueness for urban Forward Sortation Areas (FSAs) by using the 2001 long form Canadian census data representing 20% of the population. We then constructed two logistic regression models to predict when the uniqueness is greater than the 5% and 20% thresholds, and validated their predictive accuracy using 10-fold cross-validation. Predictor variables included the population size of the FSA and the maximum number of possible values on the quasi-identifiers (the number of equivalence classes).

**Results:**

All model parameters were significant and the models had very high prediction accuracy, with specificity above 0.9, and sensitivity at 0.87 and 0.74 for the 5% and 20% threshold models respectively. The application of the models was illustrated with an analysis of the Ontario newborn registry and an emergency department dataset. At the higher thresholds considerably fewer records compared to the 0% threshold would be considered to be in small areas and therefore undergo disclosure control actions. We have also included concrete guidance for data custodians in deciding which one of the three uniqueness thresholds to use (0%, 5%, 20%), depending on the mitigating controls that the data recipients have in place, the potential invasion of privacy if the data is disclosed, and the motives and capacity of the data recipient to re-identify the data.

**Conclusion:**

The models we developed can be used to manage the re-identification risk from small geographic areas. Being able to choose among three possible thresholds, a data custodian can adjust the definition of "small geographic area" to the nature of the data and recipient.

## Background

The disclosure and use of health data for secondary purposes, such as research, public health, marketing, and quality improvement, is increasing [1-6]. In many instances it is impossible or impractical to obtain the consent of the patients ex post facto for such purposes. But if the data are de-identified then there is no legislative requirement to obtain consent.

The inclusion of geographic information in health datasets is critical for many analyses [7-15]. However, the inclusion of geographic details in a dataset also makes it much easier to re-identify patients [16-18]. This is exemplified by a recent Canadian federal court decision which noted that the inclusion of an individual's province of residence in an adverse drug event dataset makes it possible to re-identify individuals [19,20].

Records from individuals living in small geographic areas tend to have a higher probability of being re-identified [21-23]. Some general heuristics for deciding when a geographic area is too small with respect to identifiability have been applied by national statistical agencies [24-29]. For example, the US Health Insurance Portability and Accountability Act (HIPAA) Privacy Rule defines a small geographic area as one having a population smaller than 20,000.

Common disclosure control actions for managing the re-identification risks from small geographic areas are to: (a) suppress records in the small geographic areas, (b) remove from the disclosed dataset some of the non-geographic variables, (c) reduce the number of response categories in the non-geographic variables (i.e., reduce their precision), or (d) aggregate the small geographic areas into larger ones. None of these options is completely satisfactory in practice. Options (a) and (b) result in the suppression of records or variables respectively. The former leads to the loss of data and hence reduces the statistical power of any analysis, and can also result in bias if the suppressed records are different in some important characteristics from the rest of the data. The latter is often difficult to implement because variables critical to the analysis of the data cannot be removed. Options (c) and (d) reduce the precision of the information in the dataset through generalization. The former generalizes the non-geographic information in the dataset which may make it difficult to detect subtle trends and relationships. The latter can reduce the ability to perform meaningful analysis and can conceal variations that would otherwise be visible at smaller geographical scales [30-35].

Given the detrimental effects of such disclosure control actions, it is important to have accurate and proportionate methods for assessing when a geographic area is too small.

The uniqueness of individuals is often used as a surrogate measure of re-identification risk [36]. An individual is unique if s/he is the only individual with a specific combination of values on their personal characteristics that are included in a dataset. There is a monotonically decreasing relationship between uniqueness and geographic area population size: uniqueness decreases as population size gets larger. A recent study developed a model to decide when a geographic area is too small based on the uniqueness of its population [37]: if uniqueness within a geographic area is approximately zero then the geographic area is not too small.

However, using zero uniqueness as a threshold for disclosure control is quite stringent and can result in excessive record or variable suppression and/or aggregation. Higher uniqueness thresholds have been found acceptable and have been applied in practice. Specifically, previous disclosures of cancer registry data have deemed thresholds of 5% and 20% population uniqueness as acceptable for public release and research use respectively [38-40].

In this paper we extend this line of work by developing models to determine whether a Forward Sortation Area (FSA - the first three characters of the Canadian postal code) is too small based on the 5% and 20% uniqueness thresholds by analyzing Canadian census data. We also provide data release risk assessment guidelines for deciding which one among the 0%, 5%, and 20% threshold models to use for disclosure control.

## Methods

Our approach was to construct models to determine if the percentage of unique records in a particular FSA was above the 5% and the 20% thresholds. These models characterize each FSA in terms of its population size, and also take into account the characteristics of the non-geographic variables in the dataset that can be used for re-identification.

### Definitions

#### Quasi-identifiers

The variables in a dataset that can be used to re-identify individuals are called the *quasi-identifiers *[41]. Examples of common quasi-identifiers are [37,42-44]: dates (such as, birth, death, admission, discharge, visit, and specimen collection), race, ethnicity, languages spoken, aboriginal status, and gender.

#### Equivalence Class

An equivalence class is defined as the group of records having a given set of values on the quasi-identifiers. For example, "50 year old male" represents the equivalence class of records with the "50" value on the age quasi-identifier and "Male" on the gender quasi-identifier. The number of records that have these two values on the quasi-identifiers is the size of the "50 year old male" equivalence class.

#### Uniqueness

The uniqueness of records in the dataset is based only on the quasi-identifiers. For example, if our quasi-identifiers are age and gender, then say, the only 90 year old female in the FSA "N3E" would be a unique record on these quasi-identifiers within that geographic area. Other sensitive variables that are not considered quasi-identifiers are not taken into account in the computation of uniqueness. If an equivalence class is of size one, then that represents a unique record.

#### Focus on the Forward Sortation Area (FSA)

The postal code is the basic geographical unit that we will use in our analysis. The postal code is frequently collected because it is readily available, and consequently, it is used as the geographical location of residence in health datasets [45-50]. The full six character postal code is often more specific than needed for many analyses. Further, in combination with other variables the full postal code would make it easy to re-identify individuals, especially in residential urban areas [43]. While there are many potential ways of aggregating geographic regions to construct larger areas for analysis [35], the FSA, a higher level in the postal code geographic hierarchy, is the unit that we considered.

### Dataset

The dataset we used comes from the 2001 Canadian census. The census has two forms: the short form and the long form. Approximately a 20% sample of the population completes the long form, and the remainder completes the short form. The long form individual level data is made available to researchers by Statistics Canada through its Research Data Centers (RDCs).

The RDC long form dataset only has geographic information at the level of the census tract. Because our desired analysis is at the FSA geographic unit, we developed a gridding methodology, described in Additional file 1, to assign the FSAs to individual records based on their census tracts. Census tracts are only defined for urban areas and do not cover Prince Edward Island (PEI). Therefore, rural FSAs and PEI were excluded from our analysis.

Table 1 contains the list of quasi-identifiers that were analyzed from the long form census file. These were selected to be representative of commonly used quasi-identifiers in health and health systems research. The table also includes the number of response categories for each quasi-identifier as they were used in our analysis.

**Table 1:** The list of quasi-identifiers that were analyzed from the census file

Variable Name in the 2001 Census RDC File	Definition	# Response categories^(*)^
SEXP	Gender	2

BRTHYR	Year of birth (from 1880 to 2001). Age: We defined age categories based on 5 year ranges.	24

HLNABDR	Language: Language spoken most often at home by the individual at the time of the census.	4

ETH1-6	Ethnic Origin: Refers to the six possible answers for the ethnic or cultural group(s) to which the respondent's ancestors belong.	26

ASRR	Aboriginal Identity: Persons identifying with at least one Aboriginal group.	8

RELIGWI	Religious denomination: Specific religious denominations, groups or bodies as well as sects, cults, or other religiously defined communities or systems of belief.	3

TOTYRSR	Total Years of Schooling: Total sum of the years (or grades) of schooling at the elementary, high school, university and college levels. Only available for individuals age 15+.	9

MARST	Marital Status (Legal)	5

TOTINC	Total income: Total money income received from all sources during the calendar year 2000 by persons 15 years of age and over. We defined categories in $15K ranges.	22

DVISMIN	Visible minority status	4

DISABIL	Activity difficulties/reductions: Combinations of one or more activity difficulties/reduction.	4

^(*) ^The number of response categories excludes non-specific responses such as missing values, not available or "other".

### Quasi-identifier Models

A quasi-identifier model consists of two or more quasi-identifiers (*qid*). To manage the scope of the analysis we consider only combinations of up to and including 5 *qids*. A total of 358 quasi-identifier models were analyzed. This results from the following approach of combining the qids.

Initially, for the 11 *qids *listed in Table 1, there are some similarities related to ethnicity and therefore they were treated as a group: HLNABDR, ETH1-6, RELIGWI, and DVISMIN. We defined a generic ethnicity variable, and whenever that generic ethnicity variable appears in a model it was replaced by one of the above four variables. Each substitution represented a different model. Thus, this gives 8 distinct *qids: *gender, age, ethnicity (generic), schooling, marital status, total income, aboriginal identity and activity difficulties.

Categorizing the 8 distinct *qids *by their utility by an intruder for re-identification gives the following two types:

• High utility to an intruder: gender, and age

• Possibly used for re-identification/sensitive: ethnicity, schooling, marital status, total income, aboriginal identity and activity difficulties

The different models were defined by the number of *qids *in the model and by having at least one sensitive *qid *included in each model.

For models including both age and gender, there are 42 models for the 8 distinct *qids *as follows:

• 5 *qids*: have age and gender and 20 combinations of 3 of the 6 sensitive qids.

• 4 *qids*: have age and gender and 15 combinations of 2 of the 6 sensitive qids.

• 3 *qids*: have age and gender and each of the 6 sensitive qids.

• 2 *qids*: have age and gender only - there is only one model.

Then substituting each of language, religion and visible minority for ethnicity gives an additional 48 models: 30 (3 × 10) models for 5 *qids *(ethnicity appears in 10 of the 20 models), 15 (3 × 5) models for 4 *qids *(ethnicity appears in 5 of the 15 models), and 3 (3 × 1) models for 3 *qids *(ethnicity appears in one of the 6 models).

The subtotal for this group of models containing both age and gender is 90 (42+48).

We repeated the above process for each *one *of age and gender in combination with the sensitive *qids*. That is there are 56 models containing:

• 5 *qids*: have age and 15 combinations of 4 of the 6 sensitive *qids*.

• 4 *qids*: have age and 20 combinations of 3 of the 6 sensitive *qids*.

• 3 *qids*: have age and 15 combinations of 2 of the 6 sensitive *qids*.

• 2 *qids*: have age and each of the 6 sensitive *qids *only.

Similarly to the previous group, by taking into account the ethnicity related variables, there are a sub-total of 134 models for this group.

Lastly, age is replaced with gender for an additional 134 models. Adding up the sub-totals gives a total number of 358 quasi-identifier models.

For each quasi-identifier model, we denote its maximum number of equivalence classes as its *MaxCombs *value. The *MaxCombs *value for any quasi-identifier model can be computed from Table 1. For example, if we consider the four quasi-identifiers: Age, Marital Status, Schooling, Religion, then there are 24 (age) × 5 (marital status) × 9 (years of schooling) × 3 (religion) = 3,240 possible values on these variables, which is the *MaxCombs *value. The *MaxCombs *values range from 6 to 718,848 across all quasi-identifier models.

### Estimating Uniqueness

There are a number of different approaches that can be used to estimate uniqueness in the population from the 20% sample.

The first study to examine uniqueness in the general population was conducted in the US by Sweeney [51]. Relying on the generalized Dirichlet drawer principle, she made inferences about uniqueness in specific geographic areas. This principle states that if *N *objects are distributed in *k *boxes, then there is at least one box containing at least  objects (i.e., the largest integer within the brackets). If *N *≤ *k *then there is at least one box with a single object (i.e., a unique).

Sweeney made the conservative assumption that if there is any unique in a particular geographic area, say an FSA, then that FSA is high risk. She then reported the percentage of individuals in high risk geographic areas. For example, if we consider a quasi-identifier model with a *MaxCombs *value of 48 (the *k *value), then any FSA with a population smaller than 48, say 15 (the *N *value), would likely have a unique individual in it, and therefore all 15 individuals would be considered at a high risk of uniqueness.

However, this approach will tend to overestimate the percentage of uniques because not all individuals in the FSA will be unique. For example, in the case above, on average, 26% of the 15 individuals would be non-unique. Furthermore, the Sweeney method does not help us with estimating if uniqueness is above 5% or 20% for a particular FSA.

An earlier study, which predicted when a geographic area is too small, was based on the zero uniqueness threshold utilizing a public use census file [37]. That study assumed that as sample uniqueness approached zero, the population uniqueness also approached zero. This assumption is not suitable for directly estimating population uniqueness at a 5% or 20% threshold.

Another approach to estimate equivalence class sizes was taken by Golle [52], where he assumed a uniform distribution of dates of birth of individuals living in a geographic area in assigning them to equivalence classes. However, that approach was driven by the author only having access to high level census tabulations, and was limited to a single variable. In our case the uniform distribution assumption cannot be justifiably extended to all of the quasi-identifiers.

For our analysis we used the individual-level Canadian census dataset. Given that the long form census dataset is a 20% sample of the Canadian population, we utilized uniqueness estimators to determine the proportion of unique records for each combination of FSA and quasi-identifier model. The reason we need to estimate population uniqueness is because sample uniqueness does not necessarily equate to population uniqueness, and we are interested in population uniqueness.

One estimator developed by Bethlehem et al. [36,53] over-estimates with small sampling fractions and under-estimates as the sampling fraction increases [54]. We therefore adopted a different estimation approach developed by Zayatz [31,55]. While this approach tends to over-estimate the number of population uniques for small sampling fractions, our 20% sampling fraction would be large enough to alleviate concerns about bias [54].

### Prediction Models

Based on the uniqueness estimate for each quasi-identifier model and FSA, two binary variables were constructed: the first is 1 if the estimated uniqueness for a particular FSA and quasi-identifier model was above 5% and zero otherwise, and the second was 1 if the estimated uniqueness was above 20% and zero otherwise.

This is illustrated in Table 2 through a series of examples. Here we have seven example FSAs, and for each one a set of quasi-identifiers (quasi-identifier model) is shown. For example, for the "K7N" we have the "age × sex" quasi-identifier model. For each FSA and quasi-identifier model combination we show the uniqueness estimate. Recall that we only have data on 20% of the population, therefore the uniqueness estimate gives us the percentage of individuals in that FSA who are unique on their quasi-identifier values. For instance, in "L6P" 16.7% of the population are unique on their gender, aboriginal status, schooling, and language spoken at home. The last two columns of the table indicate whether the estimated uniqueness is greater than 5% and greater than 20% respectively. Such a table was constructed for all FSAs and for all quasi-identifier models. This table had 342,606 rows.

**Table 2:** Example uniqueness estimates, *POP *and *MaxCombs *values for some FSA and quasi-identifier combinations.

Example of Uniqueness Estimates for FSA and Quasi-identifier Model Combinations					
**ID**	**FSA**	**Quasi- Identifiers**	**Uniqueness ()**	**>5%**	**>20%**

1	K7N	Age, Sex	0%	N	N

2	M2K	Age, Aboriginal, Religion	1.7%	N	N

3	K1A	Sex, Marital Status, Language	14.3%	Y	N

4	L6P	Sex, Aboriginal, Schooling, Language	16.7%	Y	N

5	H3T	Age, Aboriginal, Income, Marital Status, Language	56.0%	Y	Y

6	L1 M	Sex, Disability, Marital Status, Schooling, Ethnicity	67.80%	Y	Y

7	K1A	Age, Disability, Income, Marital Status, Schooling	94.70%	Y	Y

We developed one binary logistic regression model [56] with the 5% binary variable (denoted by *I*_05_) as the response variable, and another with the 20% binary variable (denoted by *I*_20_) as the response variable. The predictor variables in this model characterize the FSA and the quasi-identifiers in the quasi-identifier model.

An FSA can be characterized by its population size, which was obtained from the census data. We denote this variable by *POP*. For example, the "K7N" FSA in Table 3 has a *POP *value of 6,228, and the "L6P" FSA has a *POP *value of 2,247. The *POP *variable ranged from 200 to 78,457.

**Table 3:** Example of what the raw data used to build the models looked like.

Example of Raw Data Used in Building the Logistic Regression Models				
**ID**	***POP***	***MaxCombs***	***I*_05_**	***I*_20_**

1	6,228	48	0	0

2	14,047	576	0	0

3	100	40	1	0

4	2,247	576	1	0

5	12,916	84,480	1	1

6	7,080	9,360	1	1

7	100	95,040	1	1

**(b) **The population uniqueness binary value is used in the logistic regression model with the other predictor variables. We used the 2001 Canadian Census population values.

In a previous study it was shown that *MaxCombs *was a good predictor of uniqueness [37]. We therefore use it to characterize the quasi-identifier model used. Table 3 includes the *MaxCombs *values for each of the quasi-identifier models in our example, as well as the response variables for the logistic regression models. The data in Table 3 are an example of the raw values that we used in building the regression models. An observation is an FSA by quasi-identifier model combination (as shown in Table 3). For example, there is one observation for the "K7N" FSA for the quasi-identifier model "age × sex".

The 5% model was defined as:

where *π*_05 _is the probability that an observation is high risk (uniqueness greater than 5%) and the *b *parameters were estimated. The logistic regression models were estimated and evaluated using SAS version 9.1. We included an interaction term in the model so that we can adjust the relationship between *MaxCombs *and uniqueness according to the population size of the FSA (instead of creating a separate model for each FSA). The 20% model was similarly constructed.

To avoid collinearity with the interaction term in the model, both predictor variables were centered [57]. Collinearity occurs when there are linear dependencies among the predictor variables, and between predictor variables and the intercept [58]. Because both *POP *and *MaxCombs *have large values, the interaction term in the logistic regression model can create overflow problems during computation. We therefore scaled the predictor variables by 10,000.

Influential observations were identified and removed [59]. As noted below, models on different subsets of the data were constructed during our evaluation. The percentage of influential observations varied from less than 0.5% to 2.2% across these models.

### Unbalanced Dataset

Our dataset was unbalanced. This means that the proportion of observations with uniqueness less than 20% was quite small, and similarly for the proportion of observations with uniqueness less than 5%. Constructing regression models with an unbalanced dataset can result in poor model fit, inaccuracy in predicting the less prevalent class, and may even impede the convergence of the numeric maximum likelihood estimation algorithms.

There are three approaches for dealing with an unbalanced dataset: (a) a down-sampling or prior correction approach reduces the number of observations so that the two classes in the logistic regression model are equal, (b) the use of weights, and (c) an alternative correction which uses the full dataset and shown to be an improvement over weighting by King and Zeng (KZ) [60]. It has been noted that the weighting approach suffers a loss in efficiency compared to an unweighted approach when the model is exact [61], and the KZ method is shown to be better than using weights [60]. We therefore built models using two approaches and compared their results: (a) re-balancing using down-sampling, and adjusting the parameter estimates accordingly [60,62,63], and (b) the KZ method [60].

### Method for Model Evaluation

**Figure 1:**
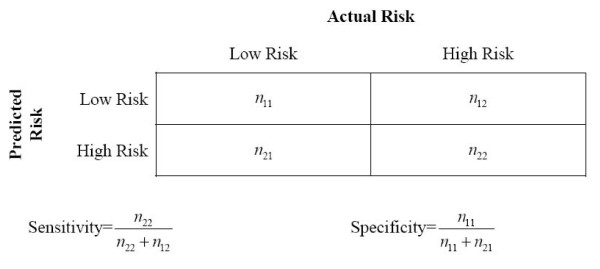
**Definition of prediction evaluation metrics**.  Low Risk means that the (predicted) percentage of unique records is below or equal to the 5% or 20% threshold.  High Rish means that the (predicted) percentage of unique records is above the 5% or 20% threshold.

We compared both methods for dealing with the unbalanced dataset problem on three values: the area under the curve (AUC) of the Receiver Operating Characteristic curve [64,65], sensitivity, and specificity. The latter two metrics are defined more precisely in Figure 1 (the AUC is based on the definitions of specificity and sensitivity).

The AUC has an intuitive interpretation: it is the estimated probability that a randomly selected observation that is above the uniqueness threshold will have a higher predicted probability from the logistic regression model than a randomly selected observation that is below the uniqueness threshold [66,67]. Sensitivity is defined as the proportion of actually high risk records (above the threshold) which were correctly predicted as such. Specificity is defined as the proportion of actually low risk records (below or equal to the threshold) which were correctly predicted as such. For computing the above metrics, if the predicted probability on the 5% threshold model was greater than 0.5 then the FSA was deemed to have a uniqueness greater than 5%. A similar predicted probability cut-off was used for the 20% threshold model.

We used 10-fold cross-validation to generate the training and test datasets, which is a generally accepted practice to evaluate prediction models in the machine learning literature [68,69]. That is, we divided the dataset used to build the logistic regression model into deciles and used one decile in turn as the test dataset, and the remaining nine deciles to build (train) the model. In the context of ten-fold cross-validation, the down-sampling and KZ methods were performed separately on the nine training deciles each time a model was estimated. All the predictions across the 10-folds were then tabulated in a 2 × 2 confusion matrix and the prediction accuracy was evaluated as illustrated in Figure 1. A confusion matrix shows the cross-tabulation of the number of observations predicted to be above/below the threshold vs. the number of observations that were actually above/below the threshold.

## Results

### Description of Canadian FSAs

Our models pertain to urban FSAs. We therefore provide a descriptive comparison of urban vs. rural FSAs in Canada.

**Figure 2:**
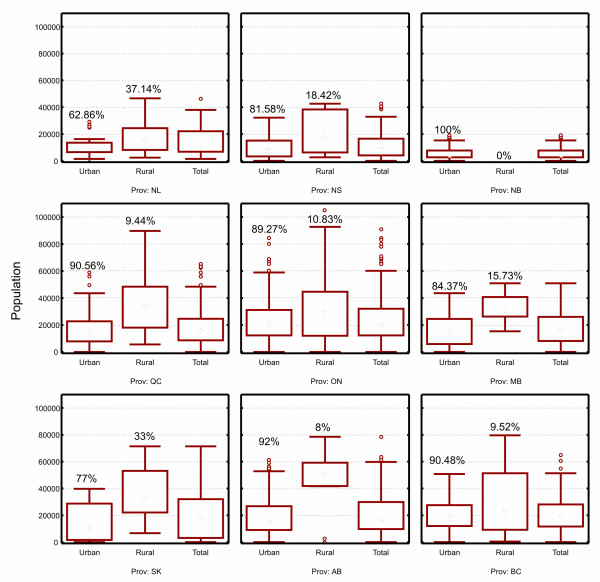
**The population sizes for urban and rural FSAs in Canadian provinces**.

**Figure 3:**
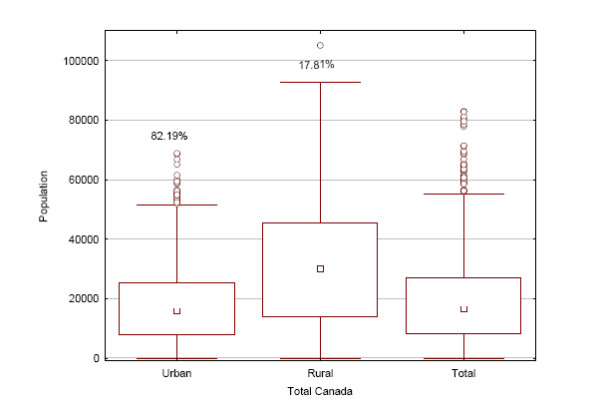
**The population sizes for urban and rural FSAs in Canada overall**.

**Figure 4:**
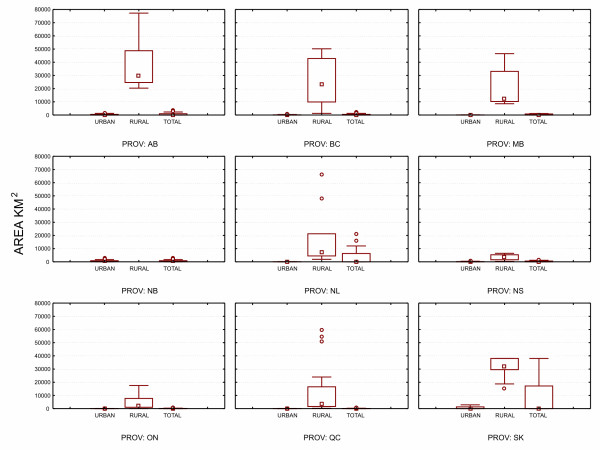
**Areas in km^2 ^for urban and rural FSAs in Canadian provinces**.

The population distribution for FSAs in the nine Canadian provinces is shown in Figure 2, and overall in Figure 3. Except for New Brunswick, rural FSAs tend to have more people living in them. The majority of the population lives in urban FSAs, except for Newfoundland, and to a lesser extent Saskatchewan, where the population is more evenly split between rural and urban FSAs. Table 4 shows the distribution of FSAs based on whether they are rural or urban. Even though they have smaller populations, the majority of FSAs are urban rather than rural. Figure 4 shows that in terms of physical size rural FSAs tend to have a considerably larger area than urban ones.

**Table 4:** Distribution of FSAs based on whether they are urban or rural.

Prov	Total Rural	Total Urban	Grand Total	%Rural	%Urban
**AB**	12	138	150	8.00%	92.00%

**BC**	18	171	189	9.52%	90.48%

**MB**	10	54	64	15.63%	84.38%

**NB**		110	110	0.00%	100.00%

**NL**	13	22	35	37.14%	62.86%

**NS**	14	62	76	18.42%	81.58%

**ON**	56	466	522	10.73%	89.27%

**QC**	39	374	413	9.44%	90.56%

**SK**	11	37	48	22.92%	77.08%

**Grand Total**	173	1434	1607	10.77%	89.23%

### Model Comparison

The two approaches for building the logistic regression models are compared in Table 5 for the two uniqueness thresholds. These results were obtained using 10-fold cross-validation. In terms of the AUC, the differences are very small and for practical purposes their predictive accuracies can be considered equivalent. The table also shows the sensitivity and specificity results using a predicted probability threshold of 0.5, which is consistent with the way that the models would be used in practice. Here we see that both modeling approaches had very similar specificity, but down-sampling had higher sensitivity for both uniqueness thresholds. Therefore, we will use the down-sampling model results in the rest of this paper.

**Table 5:** Comparison of unbalanced data modeling methods.

Model Evaluation for the 5% Uniqueness Threshold			
	**AUC**	**Sensitivity**	**Specificity**

**Down-Sampling**	0.9849	0.87	0.996

**KZ**	0.9849	0.449	0.992

**Model Evaluation for the 20% Uniqueness Threshold**			

	AUC**	**Sensitivity**	**Specificity**

**Down-Sampling**	0.947	0.74	0.98

**KZ**	0.949	0.59	0.949

**We tested the difference between the AUC values, and the difference was statistically significant between the two methods only for 20% uniqueness at an alpha level of 0.05

### Model Results

Both models had a significant goodness of fit (p < 0.001) [56]. The model parameters are shown in Table 6. All model parameters are significant, including the interaction term.

**Table 6:** Logistic regression model results for the 5% and 20% thresholds using down-sampling.

Logistic Regression Model for 5% Threshold				
	**Intercept**	***POP***	***MaxCombs***	***POP *× *MaxCombs***

**Coefficient**	779.1	-37.35	137.8	-6.5

**95% CI**	(744, 815.5)	(-60.46, -13.72)	(131.6, 144.2)	(-10.61, -2.36)

**p-value**	<0.0001	<0.0017	<0.001	0.0019

**Logistic Regression Model for 20% Threshold**				

	**Intercept**	***POP***	***MaxCombs***	***POP *× *MaxCombs***

**Coefficient**	63.3	-6	11.8	-1

**95% CI**	(61.85, 64.74)	(-6.83, -5.16)	(11.59, 12.1)	(-1.16, -0.86)

**p-value**	<0.0001	<0.0001	<0.0001	<0.0001

## Discussion

### Using the Models

In this paper we developed models to predict whether the population in a geographic area has uniqueness above the 5% and 20% thresholds using data from the Canadian census. We also demonstrated that the prediction models are sufficiently accurate to meet the risk and utility needs of data custodians and data recipients respectively. The areal unit that we studied was the urban FSA.

The logistic regression models can be used to determine whether or not the FSAs in actual datasets are too small. The *MaxCombs *value is computed based on the quasi-identifiers in the dataset. For each FSA, its population value can be determined from the Statistics Canada population tables. With these two values we can predict the probability that the percentage of uniques is above the 5% or 20% uniqueness thresholds. If the predicted probability is above 0.5, then disclosure control actions are necessary. For example, records in that FSA must be suppressed or combined with another FSA in the dataset. Alternatively, some variables may need to be removed or generalized to reduce the *MaxCombs *value.

Because the predictor variables in the models were centred and scaled, this also has to be done when using the models for actual prediction. Let the *MaxCombs *value for a particular dataset be denoted by *M*. We index the FSAs in a dataset by *j*. Let the population size for a particular FSA in the dataset be denoted by *S*_*j*_.

We have the centered and scaled *MaxCombs *value:(1)

and the centered and scaled population size value:(2)

Then an FSA is considered to be high risk under the 5% threshold if the following condition is true:(3)

and an FSA is considered to be high risk under the 20% threshold if the following condition is true:(4)

For the FSAs that are flagged through equations (3) or (4) then one should apply disclosure control actions.

### Generalization of Models

There are two types of generalizations for these models: generalization to other quasi-identifiers and generalizations to other urban areal units apart from the FSA.

Our results indicate that *MaxCombs *is a very good predictor of uniqueness. The value of *MaxCombs *does not care what type of quasi-identifiers we have - it is only affected by the number of response categories in the quasi-identifiers. A previous study has shown that taking into account the distribution of the quasi-identifiers using an entropy metric did not result in any improvement in the prediction of uniqueness [37]. One explanation for this is that we have a ceiling effect: the prediction accuracy is quite high already that the addition of distribution information cannot make a significant improvement. Consequently, a strong case can be made that the models can be used with other demographic quasi-identifiers even if they are not explicitly represented in the census dataset, and if the *MaxCombs *is within the range used in our study.

Another question is whether there is a basis for generalizing the results to other urban areal units, for example, full postal codes (which are subsets of FSAs) or regions (which are aggregates of FSAs) ? Given that the prediction models are quite accurate using only the population size as a characteristic of the area, then there is no a priori reason not to be able to apply the models to other areas as long as their population sizes are within the range used for our models and that they are for urban Canadian areas.

### Application of Models

We applied the models to evaluate whether the FSA sizes were appropriate on two data sets: the newborn registry of Ontario (Niday) and emergency department data from the children's hospital in Ottawa. In this application we assume that the disclosure control action taken is the suppression of records in small FSAs.

The Niday registry captures information about all births in the province. We used a data extract for all births during 2005-2007 fiscal years. There were 164,272 usable records in the registry during that period. The quasi-identifiers that were considered were: mother's age, baby's month and year of birth, baby's gender, and the primary language spoken at home.

The proportion of records in the Niday registry that would have to be suppressed under each of the three thresholds was computed. The results of this analysis are shown in Table 7. For example, under the 0% uniqueness threshold, 85% of the dataset would be in FSAs that are deemed too small. These small FSAs would have to be suppressed. As can be seen, there is a pronounced difference between using the 0% threshold and the others, with far less data having to be suppressed for the 5% and 20% thresholds. These results demonstrate that, where the risk profile is acceptably low, using a higher threshold can result in significantly more data being made available.

**Table 7:** The percentage of Niday and emergency department records that would have to be suppressed because they are high risk for each of the uniqueness thresholds.

	0% Threshold	5% Threshold	20% Threshold
**Niday**	85%	77%	0%

**Emergency Dept**.	93%	54%	0%

Using a similar approach, Table 7 also shows the results for the emergency department data for all presentations from 1^st ^July 2008 to 1^st ^June 2009, which consisted of 107,269 records. This data consists of date of presentation and the age of patient. With the 0% threshold 93% of the records would have to be suppressed, whereas only 54% would be suppressed for the 5% threshold, and none for the 20% threshold.

### Selection of Threshold

An important decision when using the above models is selecting which of the three uniqueness threshold to use: 0%, 5%, or 20%. The most stringent uniqueness threshold of zero percent would be appropriate for datasets that are released to the public. This threshold would result in the most suppression and aggregation. The most permissive 20% threshold can be used when disclosing data to trusted recipients where the overall risks are quite low. This larger threshold would result in the least suppression and aggregation.

To assist with deciding which of the thresholds is most appropriate under a broad set of conditions, three general criteria have been proposed in the context of secondary use [70-72]:

• Mitigating controls that are in place at the data recipient's organization.

Mitigating controls evaluate the extent to which the data recipient has good security and privacy practices in place. A recent checklist can be used for evaluating the extent to which mitigating controls have been implemented [73]. The fewer security and privacy practices that the data recipient has in place, the lower the threshold that should be used.

• The extent to which a disclosure (inadvertent or otherwise) constitutes an invasion of privacy for the patients.

Additional file 2 contains a set of items that have been developed based on the literature to evaluate the invasion-of-privacy construct [74-79]. This set of items was subsequently reviewed by a panel of 12 Canadian privacy experts for completeness, redundancy, and clarity. The greater the risk of an invasion of privacy, the lower the threshold that should be used.

• The extent to which the data recipient is motivated and capable of re-identifying the data.

Additional file 2 contains a set of items that have been developed based on the literature to evaluate the motives and capacity construct [80-83]. This construct captures the fact that some data recipients can be trusted more than others (e.g., researchers vs. making data available to the general public). The set of items was subsequently reviewed by a panel of 12 Canadian privacy experts for completeness, redundancy, and clarity. The greater the risk that the data recipient is motivated and has the capacity to re-identify the database, the lower the threshold that should be used.

Admittedly, the use of these checklists remains qualitative, but they do provide a starting point for deciding what an appropriate threshold should be.

### Limitations

The FSAs that were included in our analysis were from urban areas in Canada. As described in Additional file 1, the reason is that the census tract information from the census file that we used is only defined for urban areas. Therefore, FSAs from rural areas were not covered. However, it should be noted that the majority of the Canadian population lives in urban areas.

Our analysis was based on data from the 2001 census. There will be changes in the population over time and therefore the models may not be an accurate reflection of uniqueness the further from 2001 we are. Future studies should replicate this research on subsequent census data (the 2006 census data was not available in the Statistics Canada RDC when we conducted this study).

We used the estimated uniqueness values as the correct values, and validated our prediction model on that basis. However, the uniqueness estimate will not be perfect and such errors will negatively affect the overall accuracy of the 5% and 20% prediction models.

The *MaxCombs *value can only be computed for quasi-identifiers with a finite number of response categories. Continues variables that are not discretized cannot be sensibly captured using our approach.

## Conclusions

Disclosure control practices for small geographic areas often result in health datasets that have significantly reduced utility. These practices include the suppression of records from individuals in small geographic areas, the aggregation of small geographic areas into larger ones, suppression of the non-geographic variables, or generalization of the non-geographic variables. Previous work has used a rather stringent definition of a small geographic area: when it has no unique individuals on the potentially identifying variables (quasi-identifiers). However, less stringent thresholds have been used in the past for the disclosure of health datasets: 5% uniqueness and 20% uniqueness.

In this paper we develop models to determine whether urban FSAs in Canada are too small by the 5% and 20% criteria by analyzing 2001 census data. We have also provided a set of concrete guidelines to help custodians decide which one these thresholds to use. Within this framework, a data custodian can manage the amount of geographic suppression or aggregation in proportion to the risks of disclosing a particular dataset.

## Competing interests

The authors declare that they have no competing interests.

## Authors' contributions

KEE designed the study, directed the data analysis, and contributed to writing the paper. AB performed the Statistics Canada RDC statistical analysis and contributed to writing the paper. PA performed the geospatial data analysis and contributed to writing the paper. AN performed the model building analysis work. MW contributed to the application of the results. JB contributed to the application of the results. TR contributed to the application of the results. All of the authors have read and approved the final manuscript.

## Pre-publication history

The pre-publication history for this paper can be accessed here:

http://www.biomedcentral.com/1472-6947/10/18/prepub

## Supplementary Material

Additional file 1 **Mapping census geography to postal geography using a gridding methodology**. Describes the methodology we used to assign a postal code to each record in the census file. Click here for file

Additional file 2 **Evaluating dimensions of risk**. Presents the validated checklists for evaluating the "invasion of privacy" and "motives and capacity" dimensions of disclosure risk. Click here for file
