# Supplementary material for: A method for managing re-identification risk from small geographic areas in Canada
Source: BMC Med Inform Decis Mak. 2010 Apr 2;10:18. doi: 10.1186/1472-6947-10-18 (PMC2858714; doi:10.1186/1472-6947-10-18)
Supplement: Additional file 1 — Mapping census geography to postal geography using a gridding methodology. Describes the methodology we used to assign a postal code to each record in the census file. [file 1472-6947-10-18-S1.PDF]

Additional File 1: Mapping Census Geography to Postal Geography Using a Gridding Methodology

Background

The smallest geographic unit provided in the census microdata file available through Statistics Canada's Research Data Centre (RDC) is the census tract (CT). CTs are only defined for census metropolitan areas and census agglomerations with urban core populations of at least 50,000 individuals. They are defined by Statistics Canada as "…small, relatively stable geographic areas that usually have a population of 2,500 to 8,000." [1]. The 2001 census contained a total of 4,798 CTs distributed over 9 provinces (no CTs are defined for the Territories or the province of PEI; see Figure 1).

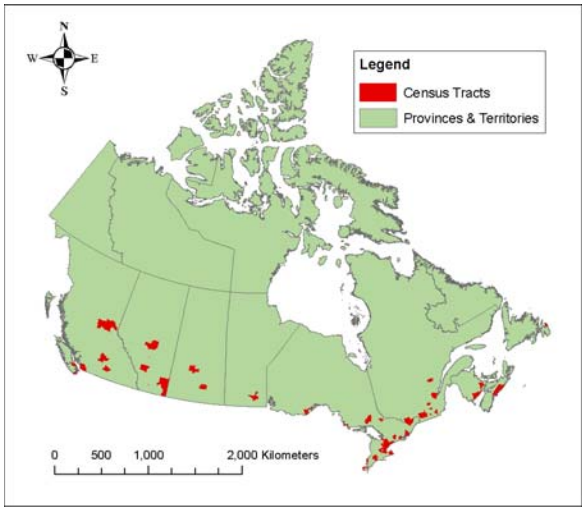

Figure 1: Distribution of 2001 census tracts across Canada.

In order to compute re-identification risk by Forward Sortation Area (FSA) in our current study, we needed to devise a method to estimate conversion between census and postal geography. A gridding methodology similar in nature to the Gridded Population of the World Project (GPW) [2] at the Center for International Earth Science Information Network at Columbia University [3] was utilized, allowing assignment of geography based on areal weighting using a population grid for Canada.

Methods

Population-based weights were assigned to CT-FSA unions based on a created population grid for all of Canada. The grid cell size was one kilometre by one kilometre, and assigned populations were based on the 2001 census profile at the Dissemination Area level (DA). This is the smallest geography at which census profile information is released by Statistics Canada [4]. Similar to the PCCF+ [5-8], these population weights were then used to randomly assign census tracts to their associated FSAs. Details of the steps taken to create the population grid are described below.

Twenty six (26) complete grids of dimensions 1554 by 546 Kilometres were created using a script in ESRI's ArcMap 9.2 [9], as specified in Table 1. This created 848,484 one kilometre square cells per grid, for a total of 22,909,068 cells covering the Canadian landmass.

Once the grids were created, the next task was to assign an estimated population to each cell. This was done using the Statistics Canada DA file [10]. First, all DA polygons identified as water were removed. A new DA shape file containing only land DAs was created. DA boundaries were then dissolved so that DAs with disparate polygons were captured within one record. Areas and perimeters were summed for each polygon to give the total DA area and perimeter. This reduced the number of records from 62,015 to 52,924, which matches the number of DAs as reported by Statistics Canada. Total population, as well as sex and age-stratified populations were extracted for all DAs across Canada, using four separate profile files (Western Canada and the Territories, Ontario, Quebec, and Atlantic Canada). Next, the 2001 DA population file was joined with the 2001 DA boundary file, to create a 2001 Canada DA boundary file containing total and sex and age stratified populations.

A "Select by attributes" function where population was not zero (0) was completed on the above file to create a new boundary file containing only DA polygons with reported populations. This further reduced the number of records to 49,153, creating a boundary file for non-water, populated DAs only. A "Select by location" function was completed on all 26 grids, for any cells that intersected the boundary file from the previous function. The resultant grids had a combined total cell count of 2,367,457.

A model was created using the ArcGIS model builder, and run for each of the 26 grids, to create grid section intersects with the 2001 DAs, FSAs and CTs. The model also calculated proportional grid sub-section areas and the corresponding population, based on underlying DA population and an assumption that the population was distributed proportionally to area within each of the geographic areas.

A summary was done by each CT-FSA combination, to create unique CT-FSA records with the corresponding sum of the calculated grid-section populations. These summed populations were then divided by the total sum of the gridded-CT population to give the proportion of the population in each CT that lay within the corresponding FSA. In essence, this creates a population-based weight for each CT-FSA combination, allowing us to randomly assign any given record within a CT to its most likely (population-weighted) FSA.

A simplified hypothetical example of the end result is given in Table 2 and Figure 2. In this example, 64.07% of the population in CT16003 is found in FSA *K2S*, and 35.93% in FSA *K2T*. For CT 16004, 49.35% of its population is in *K2R*, 19.48% in *K2S* and 31.17% in K2T. This reduces the table to five rows, with a population-based weight for each unique CT-FSA combination. If, for example, there were then 28 records from the microdata file falling in CT 16003, 18 (~65.86%) would be allocated to *K2S*, and 10 (~34.14%) to *K2T*.

Grid Section	x	y	rows	columns	# Cells	# Cells (DA-clipped)	# Cells (populated DA-clipped)
00	-2341699	310266	1554	546	848,484	147,282	95,225
01	-1795699	310266	1554	546	848,484	323,759	292,052
02	-1249699	310266	1554	546	848,484	400,335	352,048
03	-703699	310266	1554	546	848,484	421,104	252,417
04	-157699	310266	1554	546	848,484	442,583	112,863
05	388301	310266	1554	546	848,484	444,187	47,006
06	934301	310266	1554	546	848,484	588,000	220,587
07	1480301	310266	1554	546	848,484	514,762	202,006
08	2026301	310266	1554	546	848,484	222,848	139,035
09	2572301	310266	1554	546	848,484	79,825	30,635
10	-2341699	1864266	1554	546	848,484	490,304	181,644
11	-1795699	1864266	1554	546	848,484	843,129	253,796
12	-1249699	1864266	1554	546	848,484	753,391	84,386
13	-703699	1864266	1554	546	848,484	749,156	802
14	-157699	1864266	1554	546	848,484	563,822	1,239
15	388301	1864266	1554	546	848,484	192,569	1,005
16	934301	1864266	1554	546	848,484	587,718	1,420
17	1480301	1864266	1554	546	848,484	342,289	683
18	2026301	1864266	1554	546	848,484	220,305	48,694
19	2572301	1864266	1554	546	848,484	55,829	25,720
20	-2341699	3418266	1554	546	848,484	21,506	0
21	-1795699	3418266	1554	546	848,484	168,942	531
22	-1249699	3418266	1554	546	848,484	135,498	686
23	-703699	3418266	1554	546	848,484	229,560	0
24	-157699	3418266	1554	546	848,484	424,214	1,101
25	388301	3418266	1554	546	848,484	258,726	210
26	934301	3418266	1554	546	848,484	26,188	160
**TOTAL**					**22,909,068**	**9,647,831**	**2,345,951**

Table 1: Canadian grid development table.

CT	FSAsa	FSAsa Pop Density (per Sq. Km.)	CT Area in FSA (Sq. Km.)	Pop	CT Pop	Weight
16003	K2S-1	50	0.95	48	128	0.3750
16003	K2S-2	25	0.56	14	128	0.1094
16003	K2S-3	42	0.48	20	128	0.1563
16003	K2T-1	20	1.23	25	128	0.1953
16003	K2T-2	56	0.37	21	128	0.1641
16004	K2R-1	37	1.03	38	77	0.4935
16004	K2S-1	42	0.36	15	77	0.1948
16004	K2T-2	56	0.42	24	77	0.3117

FSAsa = FSA sub-area
Pop = Population

Table 2: Simplified hypothetical example of the weighted association between CTs and FSAs.

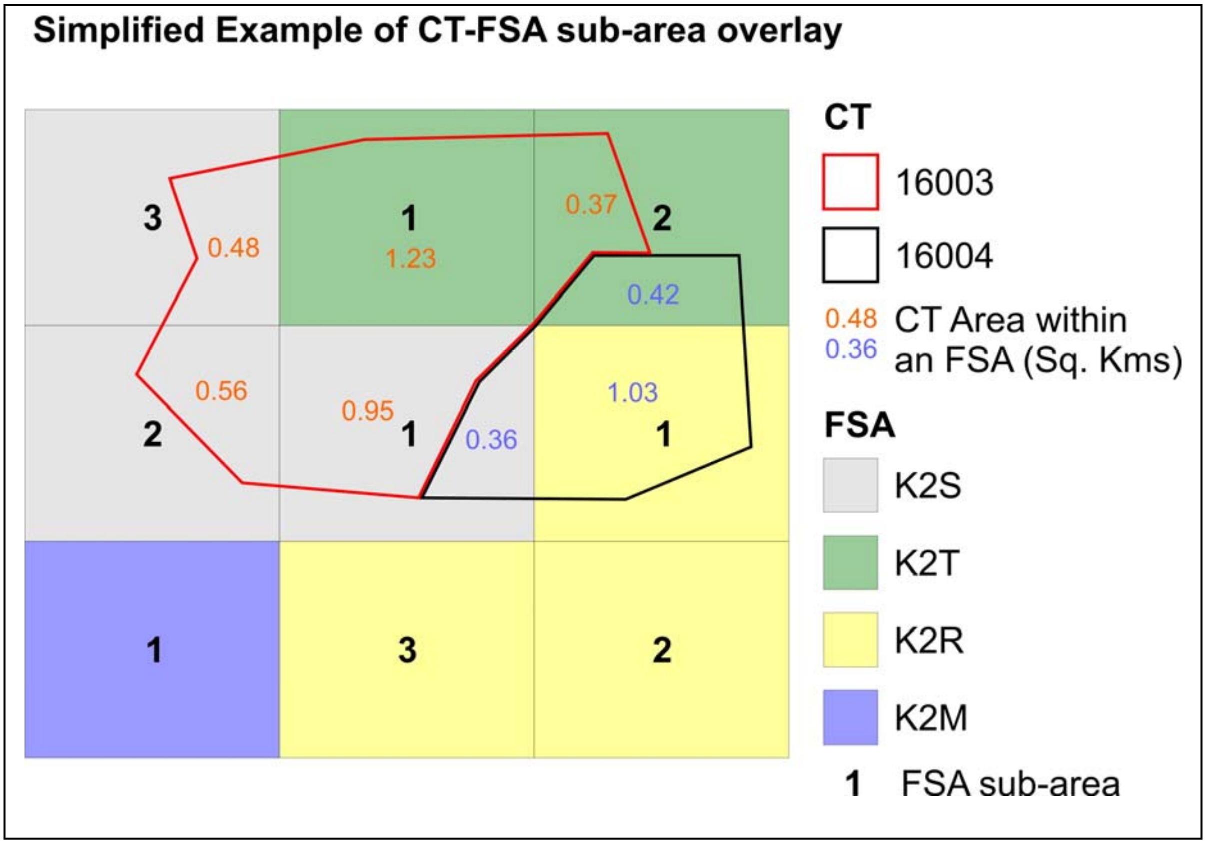

Figure 2: Example CT-FSA sub-area overlay to illustrate the hypothetical example.

Results

The CT population assignments based on the gridding methodology proved to be very similar to the 2001 Statistics Canada Census Tract population profile (Table 3). The mean difference between the populations was 3.45 individuals, with a standard deviation of 48.96 individuals (median was 0). A graphical representation of the distribution of the population differences, by census tracts, is given in Figure 3.

	2001 Statistics Canada Population Profile<br>Census Tract	Canada Population Grid Project<br>Census Tract
**Total n**	4757	4757
**Mean population**	4413.99	4410.54
**Standard Deviation**	1911.77	1911.33
**Minimum population**	40	0
**Median population**	4290	4287
**Maximum population**	20635	20636

Table 3: Census tract population comparison between created population grid and 2001 census profile.

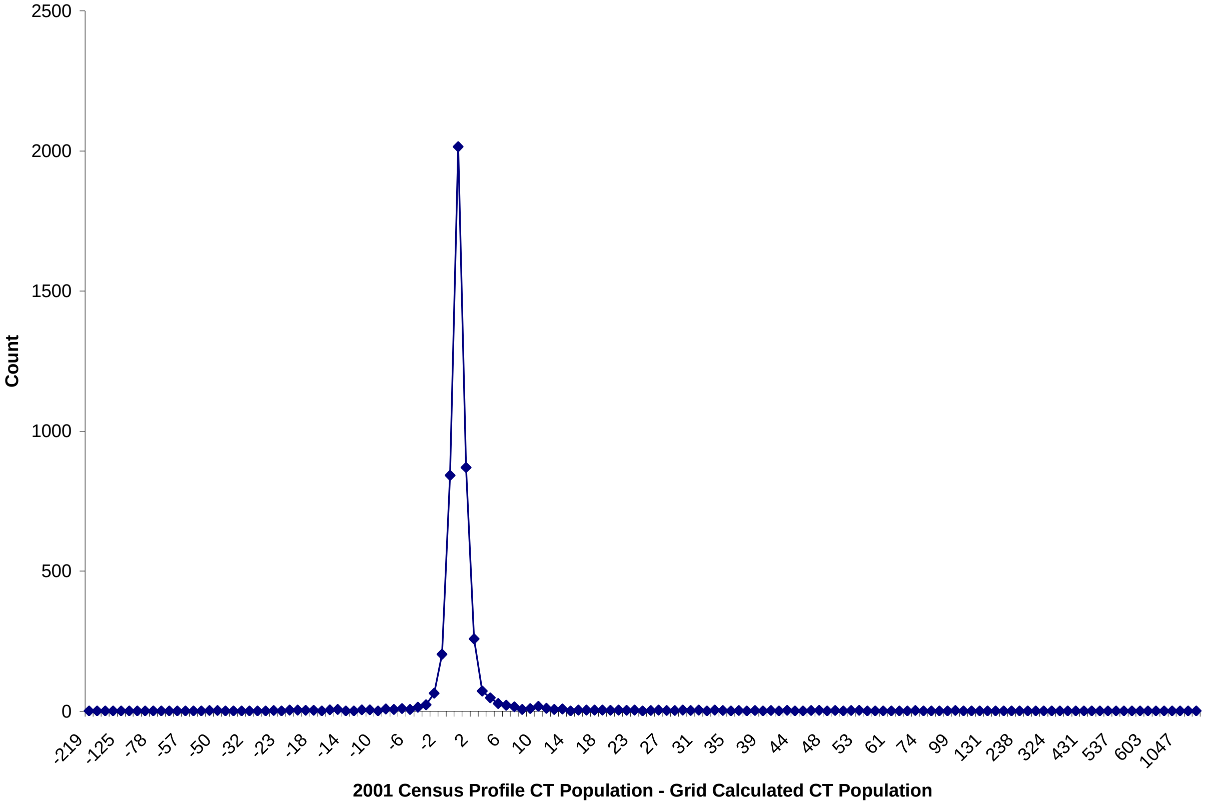

Figure 3: Distribution of Census Tract Population Difference between Grid-Calculated Population and 2001 Census Profile.

Provincial analyses also showed a high concordance between the CT populations using the gridding methodology as compared to the 2001 Statistics Canada Census Tract population profile (Table 4). The greatest differences were in New Brunswick (mean difference = 6.97 individuals, standard deviation = 75.26 individuals) and Alberta (mean difference = 6.75 individuals, standard deviation = 81.67 individuals).

	NL	NS	NB	QC	ON	MB	SK	AB	BC
**N**	45	85	70	1246	2001	164	101	449	596
**Mean**	3.71	2.6	6.97	1.55	3.68	2.93	-1.18	6.75	4.79
**Std Dev**	12.01	19.37	75.26	26.19	51.38	27.14	37.86	81.67	51.38
**Median**	0	0	0	0	0	0	0	0	0

Table 4: Provincial differences between Profile and grid CT populations.

Conclusions

The population grid created in this study provides a means for linking census geography to postal geography in Canada. While creating population grids in and of itself is not a novel idea, the created grid in this project allows the mapping of census geography to postal geography, based on population weights. The procedure assumes a uniform population distribution within the geography being used. However, since CTs only occur in highly populated urban areas, this was felt to be an appropriate assumption. A similar assumption would not hold in rural or less densely populated areas, and this technique would therefore not be appropriate. However, it could be utilized, and further refined, by incorporating additional information, such as ecumene areas, satellite imagery for residential and inhabited areas, address data, etc.

References

1. Statistics Canada. *Cartographic boundary files: 2001 census*. 2002.
2. Yetman G, Deichmann U, Balk D. *Creating a global grid of human population*. Available from: [http://gis.esri.com/library/userconf/proc00/professional/papers/PAP552/p552.htm]. Archived at: [http://www.webcitation.org/5jxHZXuVB].
3. *Center for International Earth Science Information Network (CIESIN)*. Available from: [http://beta.sedac.ciesin.columbia.edu].
4. Statistics Canada. *Profile of all levels of geography in Canada, 2001 census*. 2003.
5. Wilkins R. *Use of postal codes and addresses in the analysis of health data.* Health Reports, 1993; 5(2):157-177.
6. Wilkins R. *More about PCCF+ (for the hard core)*. 2005; Public Health Agency of Canada.
7. Statistics Canada. *Postal Code Conversion File (PCCF), Reference Guide*. 2006.
8. Mechanda K, Puderer H. *How postal codes map to geographic areas*. 2007; Statistics Canada.
9. Nicholas R. *ESRI Support Center: Create a grid polygon shapefile (FISHNET)*. 2003; Available from: [http://arcscripts.esri.com/details.asp?dbid=12807].

10. Statistics Canada. *Dissemination Areas Cartographic Boundary Files (Geography Products: Spatial Information Products, 2001 Census)*. 2002.
