# Supplementary material for: A method for managing re-identification risk from small geographic areas in Canada
Source: BMC Med Inform Decis Mak. 2010 Apr 2;10:18. doi: 10.1186/1472-6947-10-18 (PMC2858714; doi:10.1186/1472-6947-10-18)
Supplement: Additional file 2 — Evaluating dimensions of risk. Presents the validated checklists for evaluating the "invasion of privacy" and "motives and capacity" dimensions of disclosure risk. [file 1472-6947-10-18-S2.PDF]

Additional file 2: Evaluating Dimensions of Risk

The purpose of this appendix is to provide a set of items that can be used by a custodian to evaluate the risk when health information is disclosed or used for secondary purposes. The specific dimensions we look at are “invasion-of-privacy” and “motives and capacity”. Some background for the context is first provided, followed by a detailed description of each item.

Background

As illustrated in Figure 1, personal information is collected from individuals. This collection can be direct or indirect through reporters. For example, in the case of an adverse drug event, a hospital or a physician may report the adverse event rather than the patient herself.

This information remains with the custodian. An example of a custodian is a hospital or a disease registry. The custodian may have collected the information for a primary purpose, such as providing a service to a customer or providing care to a patient, or explicitly for a secondary purpose, such as a prospective diabetes registry.

A custodian may disclose personal information to another custodian. For example, a hospital may disclose personal health information to a public health agency. In such a case, the information is not coming directly from a patient but indirectly through one (or possibly more) custodians.

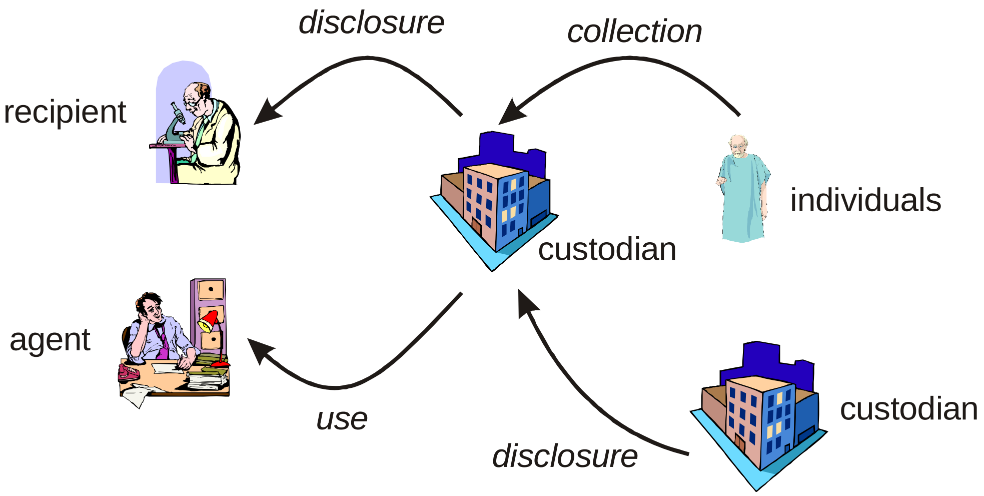

Figure 1: Basic data flow during a disclosure or use of personal information for secondary purposes.

An agent of the custodian may *use* the information for a secondary purpose. An agent is broadly defined as a person who acts on behalf of the custodian in respect of the personal information for the purposes of the custodian. For example, an analyst employed by a hospital to produce reports on resource utilization would be an agent. There is generally no legislative requirement to de-identify information that an agent uses and no requirement to obtain additional consent from the individuals/patients for such uses.

The custodian may also get a request to *disclose* the information to a recipient (or another custodian, but we will subsume that under recipient) for some secondary purpose. The recipient can be an individual (e.g., a researcher), or an organization (e.g., a pharmaceutical company or a public health agency). The recipient can also be internal or external to the custodian. For example, a researcher may be based within a hospital or can be an external researcher at a university or a government department requesting the information from the hospital.

Some disclosures are mandatory and some are discretionary to the custodian. An example of a mandatory disclosure is reporting communicable diseases or reporting gunshot wounds in some jurisdictions. In these situations the disclosure of personal information to a particular recipient is required.

Otherwise, there are different types of recipients and purposes where disclosures of personal information are discretionary. However, the conditions for discretionary disclosure do vary. There are a set of permitted disclosures in privacy legislation where personal information may be disclosed without consent, for example, disclosures for research and disclosures for planning and improving the health system.

Other discretionary disclosures that are not explicitly permitted in legislation require that either consent be obtained from the individuals/patients or that the information be de-identified. For example, the disclosure of personal health information (PHI) to a pharmaceutical company requires that consent be obtained or that the information is deemed to be de-identified.

Therefore, to summarize, there are four scenarios to consider:

A. It is mandatory to provide personal information to a recipient (usually external to the custodian), and no consent is required.

B. Personal information is used by an agent without consent.

C. It is permitted by legislation to provide personal information to a recipient without consent (either internal or external to the custodian) under the discretion of the custodian.

D. The custodian *must* de-identify the information *or* obtain consent before disclosing the data to the recipient.

The need for de-identification of the information under each of the above scenarios will vary. This is discussed further below.

The Need for De-identification

In three out of the above four scenarios where data is used or disclosed by a custodian, a strong case can be made for de-identification. Below we consider each in turn.

Scenario A: Mandatory Disclosures

Disclosures under this scenario are outside our scope since they do not require any de-identification.

Scenario B: Uses by an Agent

While agents are permitted to access personal information, if that is not necessary to perform their functions then it may be better to de-identify that information to minimize the consequences of a breach. The reason would be to mitigate risks due to data breaches, whose frequency has been increasing rapidly.

For example, consider a hospital network that has developed a system to provide its patients web access to its electronic health records. The hospital has sub-contracted the work to perform quality control for this software to a testing company across town. The testing company needs realistic patient data to test the software, for example, to make sure that the software can handle large volumes of patient records, that it displays the correct information to each patient, and so on. The testing company would be considered an agent of the hospital, so it can obtain identifiable patient records without consent, and use these records for testing. Giving the testing company PHI potentially exposes the hospital to additional risk if there is an inadvertent disclosure of this data (e.g., a breach at the testing company's site). It is always preferable from a risk management perspective to minimize the number of people who have access to PHI, and making that information available to the whole test team should be avoided if possible. Therefore in cases where there is a legitimate use of the PHI, one should still consider using de-identification techniques even if this is not a legal or regulatory requirement.

Scenario C: Permitted Disclosures

In some cases, even though the disclosure of identifiable health information is permitted by legislation, the custodian may consider de-identification anyway. This, of course, makes sense only if the purpose can be satisfied without having identifiable information. In practice, achieving many purposes does not require identifiable information. A good example of that is in the context of research.

A Research Ethics Board (REB) determines whether custodians can disclose personal information to researchers, and whether that information needs to be de-identified. REBs have total discretion to make that decision.

In practice, most REBs will require that either consent from the patients be sought if the information needs to be identifiable or they will require that the disclosed information is adequately de-identified [2]. However, because of the discretionary nature of this type of disclosure, they may allow identifiable information to be disclosed without consent.

For example, consider the situation where a researcher is collecting clinical information from electronic health records (EHRs) and wants to link it with data in a provincial administrative database. The linking will not work if the EHR data is de-identified. In that case the REB may allow identifiable information to be disclosed for the purpose of linking without requiring the consent of the patients.

Scenario D: De-identification vs. Consent

In this scenario the custodian does not have the option to disclose identifiable information without consent. However, there will be situations where obtaining consent is not possible or practical. For example, in a health research context, making contact with a patient to obtain consent may reveal the individual's condition to others against their wishes, the size of the population represented in the data may be too large to obtain consent from everyone, many patients may have relocated or died, there may be a lack of existing or continuing relationship with the patients to go back and obtain consent, there may be a risk of inflicting psychological, social or other harm by contacting individuals and/or their families in delicate circumstances, it may be difficult to contact individuals through advertisements and other public notices, and undue hardship may be caused by the additional financial, material, human, organizational or other resources required to obtain consent. In those instances, the disclosure of personal information would not be permissible and de-identification provides the only practical option for disclosure (assuming that the purpose can be achieved with the de-identified information). There is no legislative requirement to obtain consent for de-identified information.

Even if obtaining consent was possible and practical, it may have a severe adverse consequence on the information's quality because individuals who consent tend to be different on many characteristics than those who do not consent (e.g., on age, gender, socioeconomic status, whether they live in rural or urban areas, religiosity, disease severity, and level of education) [3]. These differences can result in biased findings when the information is analyzed or used. In such circumstances a strong case can be made for not seeking consent and de-identifying the information instead (again, assuming that the de-identified information will achieve the purpose of the disclosure).

Consider an example where a hospital is disclosing prescription data to a commercial data broker. It is not practical to obtain consent from the patients for this disclosure. Just the cost of administering the additional consent forms for admitted patients would be difficult to justify, and it would be difficult to do so retroactively for historical data. Therefore, the hospital would have to de-identify the prescription data before disclosure.

Items

The following items along the two dimensions of risk, "invasion-of-privacy" and "motives and capacity", can be used to decide the risk profile of the disclosure or use.

Subjectivity

The items that are defined below can be subjective in some cases. The custodian would need to define their own standards for interpreting them to reduce the subjectivity. For example, the item on the level of detail in the health information that is used/disclosed requires that the custodian define that given the nature of their data and apply it consistently across all disclosures/uses.

Layout

The following is an explanation of the layout of each item that is used to evaluate invasion-of-privacy and motives and capacity:

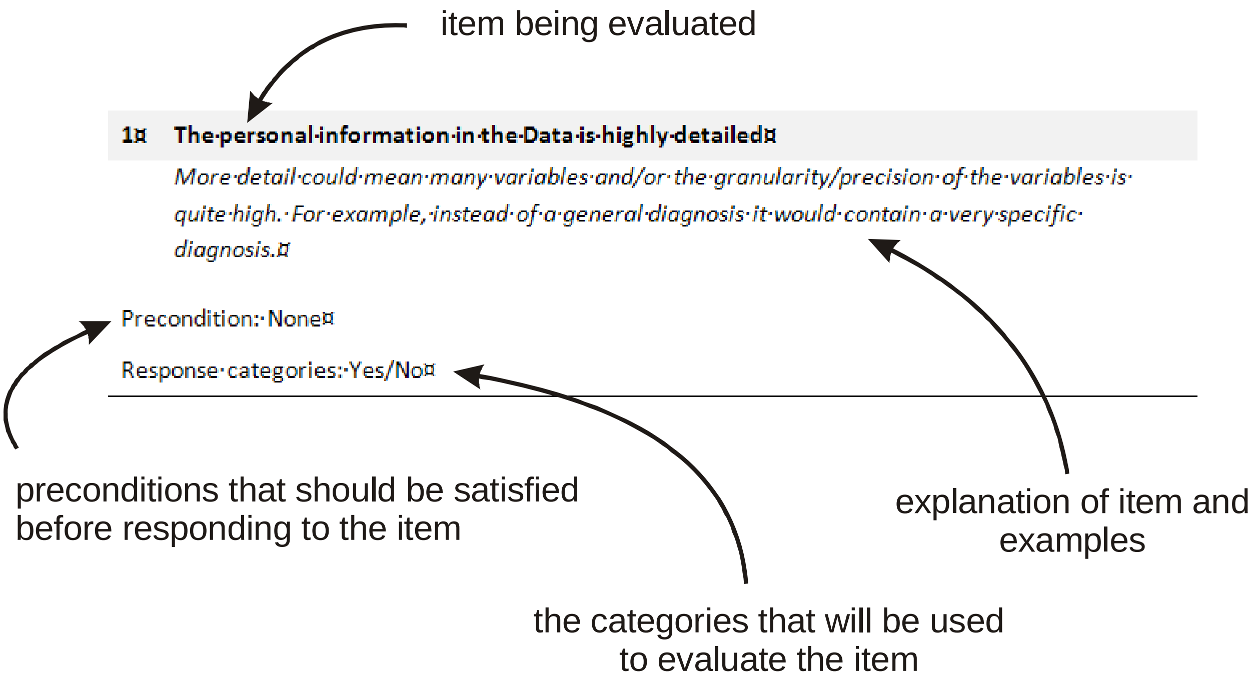

Assessing Invasion-of-Privacy

The objective of this section is to define a way to measure the invasion-of-privacy construct. There are degrees of invasion-of-privacy, and the items in this section determine that degree. By measuring the extent of the potential invasion-of-privacy, it will be possible for the custodian to decide how much de-identification needs to be done. For example, if data on a stigmatized disease is disclosed to a recipient, then that would score higher on invasion-of-privacy than disclosing data on common allergies. In both cases there would be an invasion-of-privacy, but in terms of degree the latter would be greater and therefore the data requires more de-identification.

Invasion-of-privacy is a hypothetical construct. In our definition of invasion-of-privacy we make two important assumptions:

1. An invasion-of-privacy can only occur if the data that is disclosed/used is identifiable. Therefore, all of the items below are based on the assumption that all of the data is identifiable by the recipient/agent. The custodian may have disclosed identifiable data, the disclosed data was de-identified and the recipient was able to re-identify it somehow, or the agent is using identifiable information provided by the custodian. When we talk about data in the context of this construct, then, we are referring to personal information or personal health information.

2. The disclosure/use will not entail going back to the patients and seeking their consent.

With the above assumptions, an invasion-of-privacy can occur under three conditions:

1. If the custodian inappropriately discloses the data to the recipient or there is an inappropriate use of the data.

2. If the recipient or agent inappropriately processes the data (e.g., in terms of the analysis performed on it).

3. There is a data breach at the recipient or agent site (whether it is deliberate or accidental).

The items below are intended to assess the different dimensions of invasion-of-privacy if any of the three conditions above are satisfied.

The custodian is expected to be able to respond to/assess all of the items below. In some cases the custodian may have to exercise their best judgment in order to respond.

It is assumed that it would be possible to make general assessments about all of the patients covered by the data, even if this is an approximation. For example, some patients may care if they have been consulted if their data is disclosed/used for secondary purposes, while others may not. However, if a nontrivial proportion of the patients would have cared then the particular item would be rated closer to the affirmative.

Definitions

**Data**	This is identifiable or potentially identifiable information. The Data can be identifiable if it explicitly contains identity information, such as names and phone numbers. The Data is potentially identifiable if it is relatively easy for the recipient or agent to assign identity to the Data. For example, if the identity information was replaced by pseudonyms and the recipient/agent is able to reverse engineer the pseudonyms because s/he has the pseudonym-to-identity mappings or can get them. Alternatively, the recipient/agent may have the power to compel the release of identity information. For example, if the Data has an IP address and the recipient is a law enforcement agency, then the agency may be able to compel the ISP to reveal the name and address associated with the IP address at the specified date and time.
**Purpose**	This is the purpose for which the recipient/agent has requested the Data.

Dimensions

The invasion-of-privacy construct has four dimensions:

- The sensitivity of the Data: the greater the sensitivity of the data the greater the invasion-of-privacy.

- The potential injury to patients from an inappropriate disclosure/use/breach/processing: the greater the potential for injury the greater the invasion of privacy.

- The appropriateness of consent for disclosing/using the Data: the less appropriate the consent the greater the invasion-of-privacy.

These are detailed further below.

A. Sensitivity of the Data

1 The personal information in the Data is highly detailed

More detail could mean many variables and/or the granularity/precision of the variables in the Data is quite high. For example, instead of a general diagnosis it would contain a very specific diagnosis. For instance, a high level diagnosis would be “disorders of the thyroid gland”, whereas a more detailed diagnosis would be “nontoxic nodular goiter”, and “absence of teeth” can be generalized to “diseases of oral cavity, salivary glands, and jaws”.

Precondition: None

Response categories: Yes/No (Yes = more invasion-of-privacy; No = less invasion-of-privacy)

---

2 The information in the Data is of a highly sensitive and personal nature

This could mean, for example, information about: sexual attitudes, practices, and orientation; use of alcohol, drugs, or other addictive substances; illegal activities; suicide; sexual abuse; sexual harassment; mental health; certain types of genetic information; and HIV status.

Information about a stigmatized disease/condition or that can adversely affect a patient’s business dealings, insurance, or employment would also be considered sensitive.

Precondition: None

Response categories: Yes/No (Yes = more invasion-of-privacy; No = less invasion-of-privacy)

---

3 The information in the Data comes from a highly sensitive context

For example, in most cases data about individuals participating in a youth employment program are less sensitive than a similar list containing names and addresses of Hepatitis C and HIV compensation victims. But the sensitivity may also be dependent on the specifics. For example, a list of customers on a newspaper carrier’s route may not be sensitive, unless the newspaper or publication being distributed indicates sexual orientation, for instance.

Precondition: None

Response categories: Yes/No (Yes = more invasion-of-privacy; No = less invasion-of-privacy)

B. Potential Injury to Patients

1 Many people would be affected if there was a Data breach or the Data was processed inappropriately by the recipient/agent

This item pertains to the number of patients covered by the data. More patients would be injured if there was, say, a breach of data on 10000 patients than a breach of data on 10 patients. In both cases it is an undesired outcome, but the former is more severe.

The new US HITECH Stimulus Package stipulates that any breach involving 500 or more than individuals must be reported to the Department of Health and Human Services. This can be used as a guide for what is considered as a large number of people.

If an inappropriate disclosure would affect a defined community (e.g., a minority group living in a particular area) then the number of people affected would be larger than the patients covered by the Data.

Precondition: None

Response categories: Yes/No (Yes = more invasion-of-privacy; No = less invasion-of-privacy)

---

2 If there was a Data breach or the Data was processed inappropriately by the recipient/agent that may cause direct and quantifiable damages and measurable injury to the patients

Damages and injury would include physical injury such as due to stalking or harassment; emotional or psychological harm; social harm such as stigmatization, humiliation, damage t reputation or relationships; financial harm, such as (medical) identity theft and financial fraud; and if the data can be used in making a decision that is detrimental to the patient, for example, a business, employment or insurance decision. The damages and injury can occur to the patient(s) themselves, their family unit, or to a defined group/community (e.g., neighborhood, minority groups, band leaders, Aboriginal people, people with disabilities).

Precondition: None

Response categories: Yes/No (Yes = more invasion-of-privacy; No = less invasion-of-privacy)

---

3 If the recipient/agent is located in a different jurisdiction, there is a possibility, for practical purposes, that the data sharing agreement will be difficult to enforce

It is assumed that there is some form of data sharing agreement between the custodian and the recipient/agent. For example, if the agent is an employee of the custodian, then there would be obligations in employment contracts. If the agent is a different company then there would be a contract between the custodian and that company. If the recipient is a researcher in a different institution, then a data sharing agreement would be signed by the recipient.

This particular item becomes relevant under the circumstances where the recipient/agent is in a different jurisdiction than the custodian, for example, in the US the PATRIOT Act compels custodians to disclose data in secret .In that case a law in a different jurisdiction effectively overrides the provisions in the data sharing agreement.

In some jurisdictions enforcing contracts in courts is difficult or exceedingly slow that for practical purposes the data sharing agreement cannot be enforced in that jurisdiction.

Precondition: None

Response categories: Yes/No (Yes = more invasion-of-privacy; No = less invasion-of-privacy)

C. Appropriateness of Consent

1 There is a provision in the relevant legislation permitting the disclosure/use of the Data without the consent of the patients

In some cases there will be legislative authority to disclose the Data without consent. For example, when the Data is being disclosed to a medical officer of health at a public health authority. But if the recipient was a commercial data broker then there is no exception allowing the disclosure without consent. In Ontario, custodians can disclose Data to Prescribed Entities without the patients' consent.

In the case of research, a Research Ethics Board (REB) is permitted in most jurisdictions to disclose the Data without consent. If the REB elects not to do so the response to this question would still be Yes.

Uses of Data by agents without consent are permitted in Canadian jurisdictions. Therefore, all subsequent items in this section pertain to disclosures only.

Precondition: None

Response categories: Yes/No (Yes = less invasion-of-privacy; No = no change in invasion-of-privacy)

---

2 The Data was unsolicited or given freely or voluntarily by the patients with little expectation of it being maintained in total confidence

This would pertain, for example, to patients posting their Data on a public web site as part of a discussion group. It is not always obvious that when patients post their Data on the web there is an expectation of privacy, but in some cases they may not understand the privacy settings or policies of the web site, or the organization running the web site may change their policy after the Data was collected in unexpected ways. Therefore, the response to this question must take into account the specific context and history of the location where the patients posted their information.

Precondition: If item C(1) is endorsed, then this item would not apply

Response categories: Yes/No (Yes = less invasion-of-privacy; No = no change in invasion-of-privacy)

---

3 The patients have provided express consent that their Data can be disclosed for this secondary Purpose when it was originally collected or at some point since then

This item refers to obtaining explicit consent from the patients (opt-in or opt-out). The consent may have been for the recipient's specific project (for example, in the case of patients consenting for the data that was collected during the provision of care to also be used for a specific research analysis), or may have been broad to encompass a class of projects that include the recipient's Purpose for processing the Data (for example, the patients consented for their data to be used for research on cardiovascular diseases, without knowing in advance what the possible research questions may be).

Precondition: If items C(1) or C(2) are endorsed, then this item would not apply

Response categories: Yes/No (Yes = less invasion-of-privacy; No = more invasion-of-privacy)

---

4 The custodian has consulted well-defined groups or communities regarding the disclosure of the Data and had a positive response

This item would be endorsed Yes if these well defined groups or communities did not raise objections to the particular disclosure/use. If they did consult and the outcome was negative, then the item is scored No.

Well defined groups or communities include neighborhood members, minority groups, band leaders, Aboriginal people, people with disabilities, consumer associations, community representatives, privacy oversight bodies, and patient advisory councils.

The assumption with this item is that a nontrivial proportion of patents care what their group/community thinks about the disclosure and that they be consulted.

Precondition: If items C(1), C(2), or C(3) are endorsed, then this item would not apply

Response categories: Yes/No (Yes = less invasion-of-privacy; No = more invasion-of-privacy)

---

5 A strategy for informing/notifying the public about potential disclosures for the recipient's secondary Purpose was in place when the data was collected or since then

The custodian may have given notice of potential disclosures for secondary purposes, for example, through well located posters at their site. The notice does not need to explicitly mention the particular recipient's Purpose, but should describe potential purposes that include the recipient's Purpose.

This is an example of obtaining implicit consent when there are no legislative exceptions and express consent was not obtained.

Precondition: If items C(1), C(2), or C(3) are endorsed, then this item would not apply

Response categories: Yes/No (Yes = less invasion-of-privacy; No = more invasion-of-privacy)

---

6 Obtaining consent from the individuals at this point is inappropriate or impractical

For example, making contact to obtain consent may reveal the individual's condition to others against their wishes, the size of the population is too large to obtain consent from everyone, many patients have relocated or died, there is a lack of existing or continuing relationship with the patients, the consent procedure itself may introduce bias, there is a risk of inflicting psychological, social or other harm by contacting individuals and/or their families in delicate circumstances, it would be difficult to contact individuals through advertisements and other public notices, and undue hardship that would be caused by the additional financial, material, human, organizational or other resources required to obtain consent.

This assessment may be contextual. For example, obtaining consent may be difficult for a researcher with limited funds, but if a large organization is requesting the data and they are expected to generate a large amount of revenue from processing the data, then the custodian may be able to convince the recipient that it is worth their while to invest in obtaining consent.

Precondition: If items C(1), C(2), C(3), C(4) or C(5) are endorsed, then this item would not apply

Response categories: Yes/No (Yes = less invasion-of-privacy; No = more invasion-of-privacy)

Assessing Motives and Capacity

The objective of this sub-section is to define a way to measure the motives and capacity construct. This construct assumes that the custodian is disclosing/using data that has gone through some kind of de-identification. Therefore, we are concerned with the motives and capacity of the recipient/agent to re-identify this data.

This construct has two dimensions: "motives" and "capacity". Since "motives" pertain to individuals, the motives dimension can be considered in terms of the staff, collaborators, or employees of the recipient/agent entity. The motive to re-identify the data implies an intentional re-identification. The capacity dimension evaluates whether the recipient/agent is able to re-identify the data, irrespective of whether the re-identification is intentional or not.

The custodian is expected to be able to respond to/assess all of the items below. In some cases the custodian may have to exercise their best judgment in order to respond as some of the items are subjective.

Definitions

**Data**	The data is assumed to have gone through some kind of de-identification before it is disclosed/used. The amount of de-identification will vary depending on the specifics of the disclosure/use.
**Purpose**	This is the purpose for which the recipient/agent has requested the Data.

A. Motives to Re-identify the Data

1 The recipient/agent has directly or indirectly worked/collaborated with the custodian in the past without incident

This item assumes that this collaboration has not resulted in any incidents where the recipient/agent processed the data in an inappropriate way or attempted to re-identify the data (i.e., it was perceived to be a successful collaboration). If the custodian has worked with the recipient/agent before then there is an empirical trust that has been built up, suggesting that the recipient is trustworthy.

Precondition: None

Response categories: Yes/No (Yes = fewer motive to re-identify; No = greater motive to re-identify)

2 The recipient/agent can potentially gain financially from re-identifying the Data

The first consideration is whether the recipient/agent is in financial distress. Although, this may be difficult to assess in practice.

Consider if the recipient/agent or his/her family/employees/collaborators may receive financial benefits from processing identifiable data. For example, a pharmaceutical company may want to contact the patients directly for marketing purposes or to recruit them in a study.

Another consideration is if the Data, once re-identified, can be useful for committing financial fraud or identity theft (e.g., the database has dates of birth and mother's maiden name).

Precondition: None

Response categories: Yes/No (Yes = greater motive to re-identify; No = fewer motive to re-identify)

3 There is possibly a non-financial reason for the recipient/agent to try to re-identify the Data

For example, there may a reason that the recipient/agent may want to embarrass the custodian by demonstrating that re-identification is possible, or say a reporter wanting to do a story about a specific identifiable person in the Data or a famous person known to be in the Data. Also, a disgruntled employee may wish to adversely affect the custodian's reputation by re-identifying a patient and making that public.

Precondition: None

Response categories: Yes/No (Yes = greater motive to re-identify; No = fewer motive to re-identify)

B. Capacity to Re-identify the Data

1 The recipient/agent has the technical expertise to attempt to re-identify the Data

Re-identification requires some basic database and statistical expertise. However, in real data sets there are missing data and data errors which would also have to be accounted for in terms of expertise. Of course an incorrect re-identification can also be problematic, but we are only concerned with a correct re-identification here.

Precondition: None

Response categories: Yes/No (Yes = greater capacity to re-identify; No = less capacity to re-identify)

2 The recipient/agent has the financial resources to attempt to re-identify the Data

Some types of re-identification require funds to get data sets to link with. Also, gathering background information about a patient in the Data who is a target of re-identification can be costly.

Precondition: None

Response categories: Yes/No (Yes = greater capacity to re-identify; No = less capacity to re-identify)

3 The recipient/agent has access to other private databases that can be linked with the Data to re-identify patients

Such private databases would only be useful if they contain the identity information about the patients. Then linkage with the de-identified database could reveal the identity of one or more patients in the Data.

Some data that can be used for linking and re-identification could be publicly available. In such a case we would consider item B(2) on the financial resources of the recipient/agent. This item pertains to private databases.

A recipient may obtain such private databases from previous disclosures by the custodian. For example, the custodian may have disclosed a particular dataset to a researcher last year, and this year the same researcher wants another dataset that can be linked to the earlier one. The recipient may also obtain a private database by colluding with someone else. For example, the researcher may arrange to link a new administrative dataset from the custodian with another researcher who has obtained a different clinical dataset from the same custodian (and the custodian would not approve for the two datasets to be linked).

An agent can also have access to data useful for linking. For example, in hospitals many staff have access to administrative data but not to clinical data. An employee can get a de-identified clinical data set and link it with the readily available administrative data set to re-identify patients in the clinical dataset.

Precondition: None

Response categories: Yes/No (Yes = greater capacity to re-identify; No = less capacity to re-identify)
